# Intermittent high oxygen influences the formation of neural retinal tissue from human embryonic stem cells

**DOI:** 10.1038/srep29944

**Published:** 2016-07-20

**Authors:** Lixiong Gao, Xi Chen, Yuxiao Zeng, Qiyou Li, Ting Zou, Siyu Chen, Qian Wu, Caiyun Fu, Haiwei Xu, Zheng Qin Yin

**Affiliations:** 1 Southwest Hospital/Southwest Eye Hospital, Third Military Medical University, Chongqing 400038, China; 2 Key Lab of Visual Damage and Regeneration & Restoration of Chongqing, Chongqing 400038, China; 3 School of Medicine, Nankai University, Tianjin 300071, China; 4 Department of Ophthalmology, Chinese People’s Liberation Army General Hospital, Beijing 100853, China

## Abstract

The vertebrate retina is a highly multilayered nervous tissue with a large diversity of cellular components. With the development of stem cell technologies, human retinas can be generated in three-dimensional (3-D) culture *in vitro*. However, understanding the factors modulating key productive processes and the way that they influence development are far from clear. Oxygen, as the most essential element participating in metabolism, is a critical factor regulating organic development. In this study, using 3-D culture of human stem cells, we examined the effect of intermittent high oxygen treatment (40% O_2_) on the formation and cellular behavior of neural retinas (NR) in the embryonic body (EB). The volume of EB and number of proliferating cells increased significantly under 40% O_2_ on day 38, 50, and 62. Additionally, the ratio of PAX6+ cells within NR was significantly increased. The neural rosettes could only develop with correct apical-basal polarity under 40% O_2_. In addition, the generation, migration and maturation of retinal ganglion cells were enhanced under 40% O_2_. All of these results illustrated that 40% O_2_ strengthened the formation of NR in EB with characteristics similar to the *in vivo* state, suggesting that the hyperoxic state facilitated the retinal development *in vitro*.

The vertebrate retina is a highly multilayered nervous tissue with a large diversity of cellular components and complex circuits^1^. Research in neurogenesis and retinogenesis is ongoing, including studies of basic neurogenesis^2^, gene networks^3^, extracellular matrix^4^, retinal stem cells^5^ and oxygen concentration^6^. However, because of the limitations in ethics and morals of human experiments, studies on those topics are limited to animals, which do not completely represent the characteristics of humans. With the development of stem cell technologies, generating tissues *in vitro* has changed developmental studies^7^.

Scientists have produced organoids, including the thyroid^8^, vascularized cardiac patch^9^, optic cup^10^,^11^, inner ear^12^ and stratified epidermis^13^, from embryonic stem cells (ESCs) in 3-D culture systems. Those organoids are generated *in vitro* through a system called the embryonic body (EB), which shares several same characteristics with early teratomas^7^. However, the behavior of EBs was more similar to that of the pregastrulating embryo^14^, which meant that EBs were the basic element for organoid development, including optic cups. Using this technology, we could repeat the developmental process and study the mechanisms of tissue formation that are difficult to understand *in vivo*. For example, the invagination of the neural retina (NR) and the hinge structure formation are both self-determined, rather than involving the pressure from the lens^10^,^15^. Additionally, *in vitro* organ development can improve our understanding of the specific cellular mechanisms of congenital diseases such as microcephaly^16^.

In the typical embryonic theory, neurogenesis occurs via neural tube formation *in vivo*. To study neurogenesis *in vitro*, many studies attempted to mimic this process. The representative induction of the neural tube from ESCs *in vitro* is the formation of neural rosettes^17^,^18^,^19^. The classic neural tube and neural rosette both consist of a pseudostratified layer of neuroepithelial cells with a characteristic apical-basal polarity, where the apical side is at the inner surface and the basal side is at the outer surface^20^. In these two neural structures, proliferating cells are located at the apical side and differentiated cells gather at the basal side^19^. These rosettes form from the continuous neuroectodermal epithelium in SFEBq culture^21^.

Oxygen, as the most common essential element participating in metabolism, is a critical factor regulating organic development. Oxygen concentration plays a vital role in the determination of cellular fate and maintenance of cellular homeostasis^22^,^23^. It is thought that low oxygen concentration retains the stemness of stem cells, while high oxygen concentration promotes the differentiation of stem cells^24^,^25^,^26^,^27^. In ESCs, high oxygen (40% O_2_) concentrations increased the proliferation and differentiation of ESCs^28^. Especially in SFEBq 3-D culture, high oxygen concentrations increased the proliferation of cells and decreased cell death within the NE^29^. Oxygen concentration also plays a crucial role in the eye and influences retinogenesis^30^,^31^. As NR tissues are the highest oxygen consumption organ in the body^6^, oxygen concentration seems to be a vital factor in NR formation. On the other hand, because generating NR in dishes is a novel technology, it is important to determine whether high oxygen environment could influence NR formation *in vitro*.

In this study, by using SFEBq culture and intermittent high oxygen treatment, the growth of EBs and the cell proliferation within the NR were discovered. The effect of high oxygen concentration on specific characteristics of NR tissue formation was also investigated. Finally, we analyzed both the biological behavior and the cellular relationship of retinal ganglion cells during retinal development *in vitro*.

## Results

### Three-dimensional culture of human ESCs (hESCs) to form EBs

**Figure 1:**
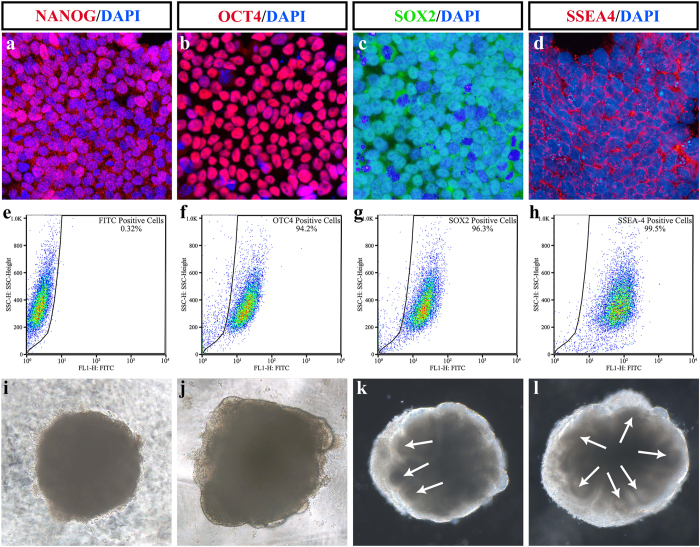
Identification of H1 human embryonic stem cell (ESC) line and early embryonic body inducement. (**a**–**d**) Immunofluorescence of ESC markers of H1 embryonic stem cell line treated with (**a**) NANOG; (**b**) OCT4; (**c**) SOX2; (**d**) SSEA4. (**e**–**h**) Fluorescence-activated cell sorting analysis of the H1 embryonic stem cell line. (**e**) Negative control. (**f**) OCT4; (**g**) SOX2; (**h**) SSEA4. (**i**–**l**) Representative image of early stage embryonic bodies. (**i**) Day 4; (**j**) Day 8; (**k**) Day 12; (**l**) Day 16. Arrows in (**k**, **l**) are pointing at the neural retina at the edges of the EBs. These data revealed that the H1 embryonic stem cell line expressed the embryonic marker. Neuroectodermal epithelia were about to emerge on day 8 and were evident from day 12.

H1 ESCs lines were first identified by ESC markers. Immunofluorescence and fluorescence-activated cell sorting analysis (FACS) showed that the H1 ESCs line expressed ESC markers, including NANOG, OCT4, SOX2 and SSEA4 (Fig. 1a–h). After stable passaging, H1 ESCs were digested into single cells. With the SFEBq culture procedure, the stem cells aggregated to form embryonic bodies from Day 1 (Fig. 1i). From Day 8, optic vesicles could be seen (Fig. 1j). After transferring EBs to the dishes from V-shaped culture plates on Day 12, optic vesicles or NR were clearly seen at the edges of the EBs (Fig. 1k, l).

### Intermittent high oxygen concentration facilitated the growth of EBs

**Figure 2:**
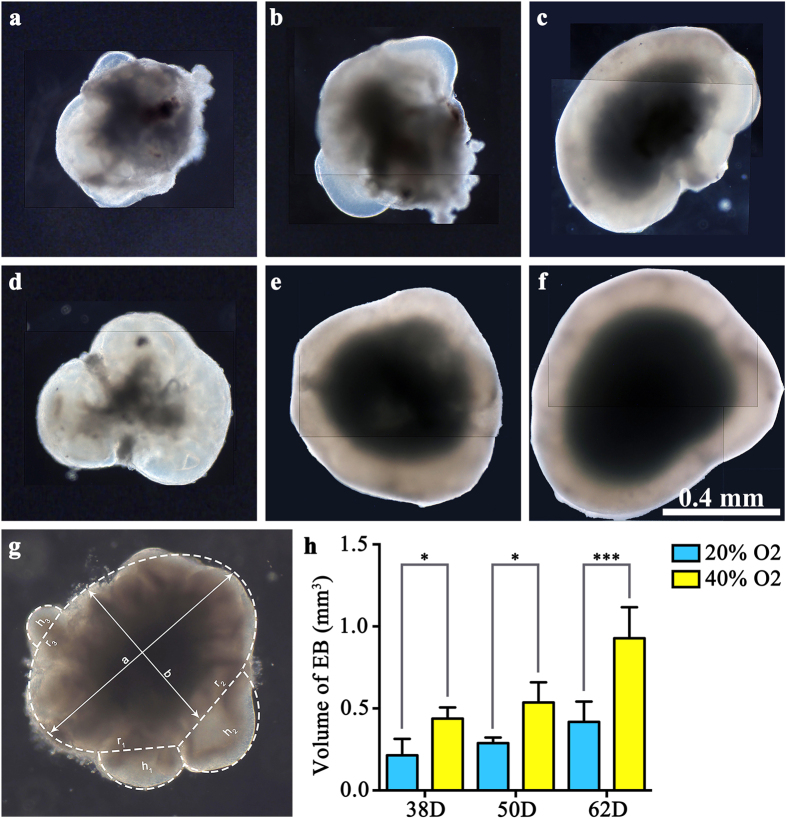
High oxygen concentration facilitated the growth of embryonic bodies. (**a**–**f**) Optical microscope images of embryonic bodies in (**a**) 38D 20% O_2_; (**b**) 50D 20% O_2_; (**c**) 62D 20% O_2_; (**d**) 32D 40% O_2_; (**e**) 52D 20% O_2_; (**f**) 62D 40% O_2_. (**g**) Representative image to measure the volume of the embryonic body. To have better resolution, figure (****b–f****) were merged together from small ones. It was divided approximately into two main parts: an ellipsoid and several spheriform segments. The ellipsoid was proximately regarded to have a long axis (**a**) and two identical short axes (**b**). The rest (n) spheriform segments were considered to have a radius (r_n_) and a height (h_n_). With integration, the volume of the embryonic body in each group was calculated and is shown in (**h**).

**Figure 3:**
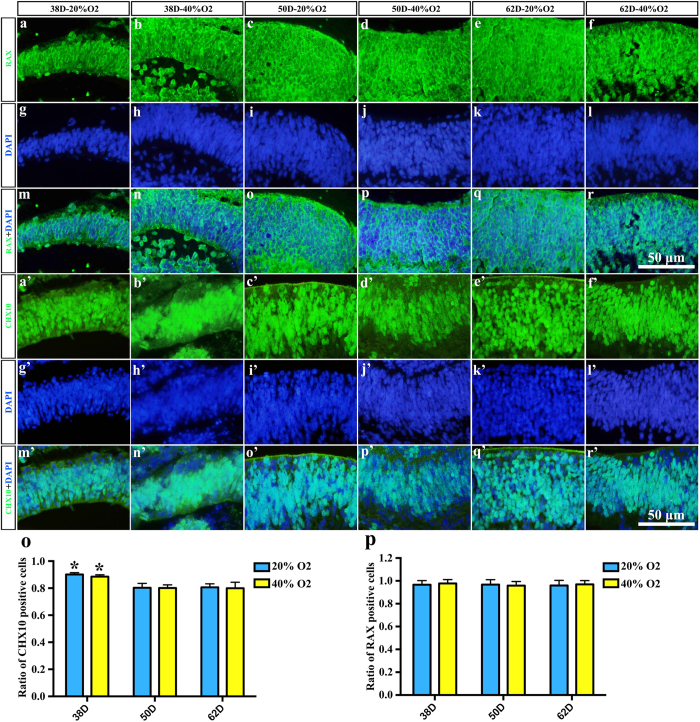
Neuroectodermal epithelium expressed the neural retina marker RAX and the retinal progenitor marker CHX10. (**a**–**f**) RAX-immunoreactive neural retina tissue of embryonic bodies in (**a**) 38D 20% O_2_; (**b**) 38D 40% O_2_; (**c**) 50D 20% O_2_; (**d**) 50D 40% O_2_; (**e**) 62D 20% O_2_; (**f**) 62D 40% O_2_. (**g**–**l**) Corresponding DAPI staining in (**a**–**f**). (**m**–**r**) Merged image of RAX and DAPI. (**a’**–**f’**) CHX10-immunoreactive neural retina tissue of embryonic bodies in (**a’**) 38D 20% O_2_; (**b’**) 38D 40% O_2_; (**c’**) 50D 20% O_2_; (**d’**) 50D 40% O_2_; (**e’**) 62D 20% O_2_; (**f’**) 62D 40% O_2_. (**g’**–**l’**) Corresponding DAPI staining in (**a’**–**f’**). (**m’**–**r’**) Merged image of CHX10 and DAPI. (**o**) statisitical analysis of CHX10 positive cells. Asterisk showed the significant difference between different time point under same oxygen concentration. (**p**) Statisitical analysis of RAX positive cells.

After 18 days’ induction and 20–44 days maturating the culture under both intermittent high oxygen concentration and normal oxygen concentration, EBs with NE positive for the neural retinal marker RAX and majorly positive for the retinal progenitor cell marker CHX10 were generated (Fig. 2a–f and Fig. 3a–r’). The CHX10 positive cells were at the largest portion on days 38 both in 20% O_2_ group and 40% O_2_ group, which significantly decreased on day 50 and day 62 (P = 0.032, P = 0.038) (Fig. 3o). EBs developed in all six groups. Using calculus, we calculated the volume of each EBs (Fig. 2g). The volume of the EBs increased significantly after treating with 40% oxygen on days 38, 50 and 62 (0.43 ± 0.10 mm^3^, 0.53 ± 0.12 mm^3^ and 0.93 ± 0.19 mm^3^, P = 0.000124) compared to the 20% oxygen-concentration culture (0.21 ± 0.10 mm^3^, 0.29 ± 0.03 mm^3^ and 0.42 ± 0.12 mm^3^, P = 0.000124). Following the post hoc test, the volume of EBs increased significantly in high oxygen conditions compared to normal oxygen conditions at the same time points (P = 0.037, P = 0.024 and P = 0.00176), which illustrated that high oxygen concentration benefited the growth of EBs (Fig. 2a–f, h). On the other hand, the EBs grew approximately 44.83% and 75.47% from day 50 to day 62 in the 20% O_2_ group and the 40% O_2_ group, respectively, which was faster than the growth from day 38 to day 50 in the 20% O_2_ group and the 40% O_2_ group (38.10% and 23.26%, respectively). This result suggests that high oxygen concentrations stimulate the growth of EBs more efficiently at a relatively late stage. Simultaneously, we determined the cell death within the central area of EBs with TUNEL staining (Supplementary method). The results of 3-D calculating showed that the number of dead cells increased gradually among all 6 groups. Though the number of dead cells in 40% O_2_ groups seemed to be higher than 20% O_2_ groups, there was no significant difference between these two groups on both days 38 and days 50. However, on days 62, the number of dead cells in 40% O_2_ group (11457399 ± 4439539) increased significantly, compared to that in 20% O_2_ group (4850692 ± 1072788) (P = 0.033) (Supplementary Fig. 1a–f, g)

### Intermittent high oxygen concentration promoted the proliferation of cells within NEs

**Figure 4:**
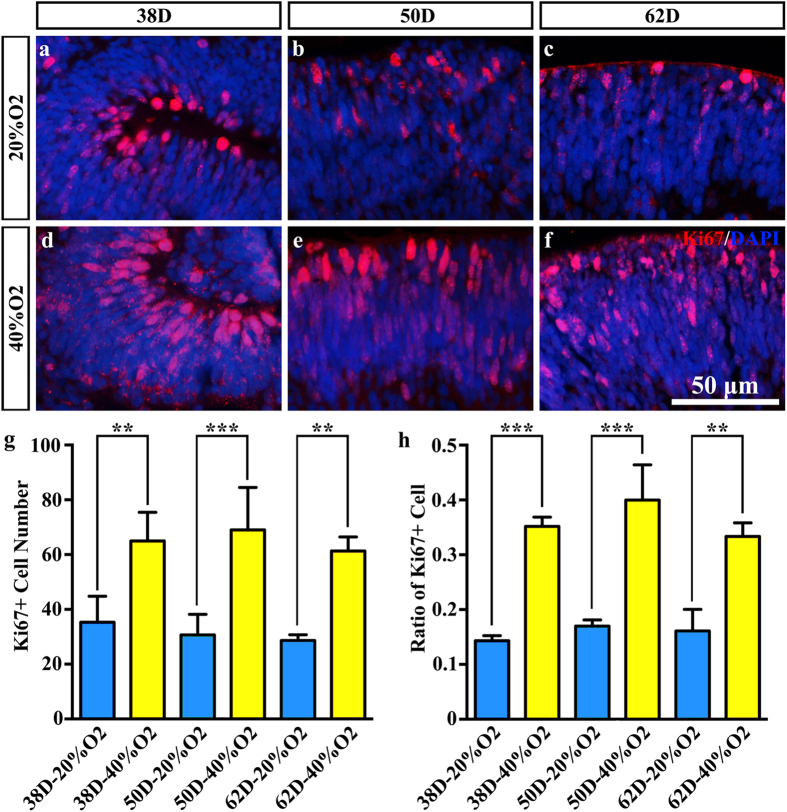
High oxygen concentration promotes cellular proliferation in the neuroectodermal epithelium (NE). (**a**–**f**) Ki67-immunoreactive proliferating cells of NE in (**a**) 38D 20% O_2_; (**b**) 50D 20% O_2_; (**c**) 62D 20% O_2_; (**d**) 32D 40% O_2_; (**e**) 52D 20% O_2_; (**f**) 62D 40% O_2_. (**g**) The number of Ki67-immunoreactive proliferating cells in the same visual field with the same magnification. (**h**) The corresponding ratio of Ki67-immunoreactive proliferating cells. In high oxygen concentration groups, the proliferation was increased compared to the normal oxygen concentration groups.

With Ki67 staining, proliferating cells within the NEs were identified^32^. The number of Ki67-positive cells within the NEs increased significantly after treating with 40% oxygen on days 38, 50 and 62 (65.00 ± 10.44, 69.00 ± 15.52 and 61.33 ± 5.13) compared to the 20% oxygen-concentration culture (35.33 ± 9.45, 31.67 ± 7.51 and 28.67 ± 2.08) (P = 0.002, P = 0.0003 and P = 0.001) (Fig. 4a–h). Additionally, the ratio of Ki67-positive cells within the NEs increased significantly after treating with 40% oxygen on days 38, 50 and 62 (35.19% ± 0.02, 39.98% ± 0.06 and 33.36% ± 0.02) compared to the 20% oxygen-concentration culture (14.33% ± 0.01, 17.00% ± 0.01 and 16.11% ± 0.04) (P = 0.00006, P = 0.00002 and P = 0.00004) (Fig. 4a–h), which corresponded to the number of Ki67-positive cells within the NEs. The proliferation peak occurred at day 50 in both the 20% O_2_ group and the 40% O_2_ group, but the cellular proliferation remained high under the high oxygen concentration. Simultaneously, the number of ectopic proliferating cells, which were not located at the apical side, was significantly higher in the high oxygen groups (Fig. 4a–h). These cells were undergoing interkinetic nuclear migration^33^,^34^ and high oxygen concentrations promote this process.

### NR grew closely to the state *in vivo* under intermittent high oxygen concentration

**Figure 5:**
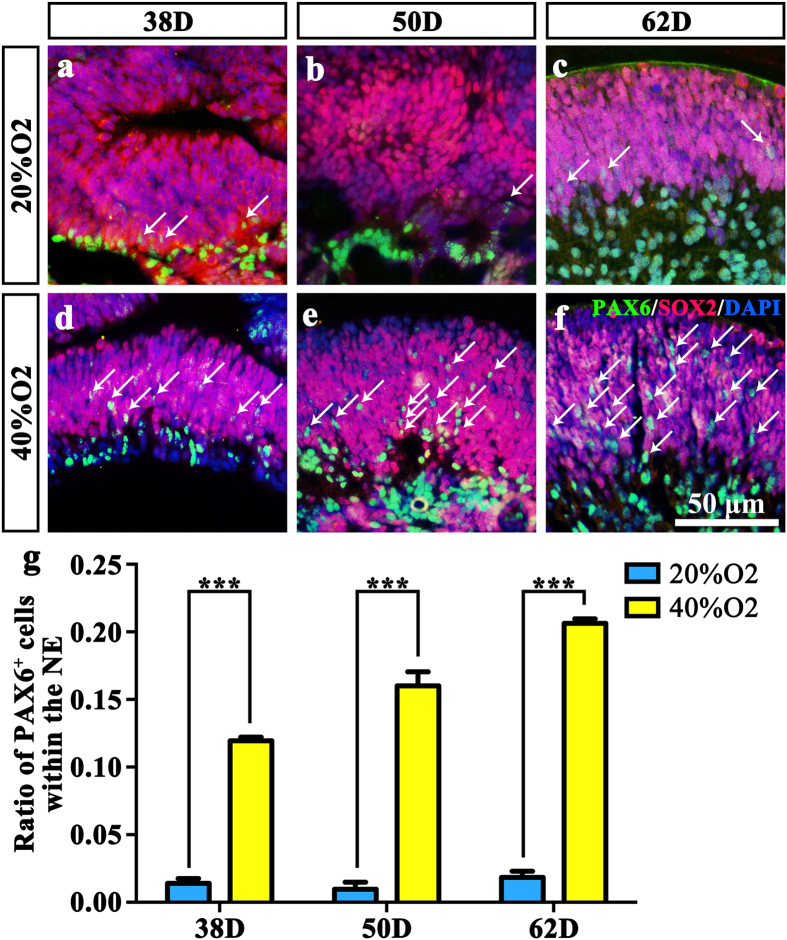
High oxygen concentration facilitates neural retinal development similar to the status *in vivo*. (**a**–**f**) SOX2 and PAX6 double-staining of NE in (**a**) 38D 20% O_2_; (**b**) 50D 20% O_2_; (**c**) 62D 20% O_2_; (**d**) 32D 40% O_2_; (**e**) 52D 20% O_2_; (**f**) 62D 40% O_2_. White arrows point at the PAX6-immunoreactive cells within the NE. (**g**) Ratio of PAX6-immunoreactive cells within the NE in 6 groups. The number of PAX6 positive cells within the NE in the high oxygen groups was much higher compared to the normal oxygen concentration groups. NE: Neuroectodermal epithelium.

Because SOX2 plays an important role in retinal development and neural cell fate determination^35^,^36^, SOX2-positive cells within the NEs were detected. In both the 20% O_2_ group and the 40% O_2_ group on days 38, 50 and 62, the NR expressed SOX2 (Fig. 5a–f). There was no difference between the groups, which indicated that oxygen concentration does not affect SOX2 expression. PAX6 also participates in retinal development^37^,^38^ and is critical to the differentiation of all retinal cells, including amacrine cells^39^. Therefore, we undertook PAX6 staining in all six groups. Since PAX6 is also expressed within the brain, double staining of PAX6 and RAX is necessary. Results showed that nearly all PAX6 positive cells also expressed the retinal progenitor cell marker RAX. There showed no difference between different oxygen concentration on days 38, 50 and 62 (P = 0.488264, P = 0.779311, P = 0.859589) (Supplementary Fig. 2a–f, g). However, the number of PAX6-positive cells within the NEs increased significantly after treating with 40% oxygen on days 38, 50 and 62 (11.95% ± 0.48%, 16.02% ± 1.79% and 20.63% ± 0.57%) compared to the 20% oxygen-concentration culture (1.40% ± 0.61%, 0.96% ± 0.92% and 1.85% ± 0.80%) (P = 0.00001, P = 0.0002 and P = 0.00001). Because PAX6-positive cells located at the middle part of the NEs differentiate into more retinal cell types, high oxygen concentration facilitates NR development more closely to the state *in vivo*.

### Oxygen concentration influenced the apical-basal neural development

**Figure 6:**
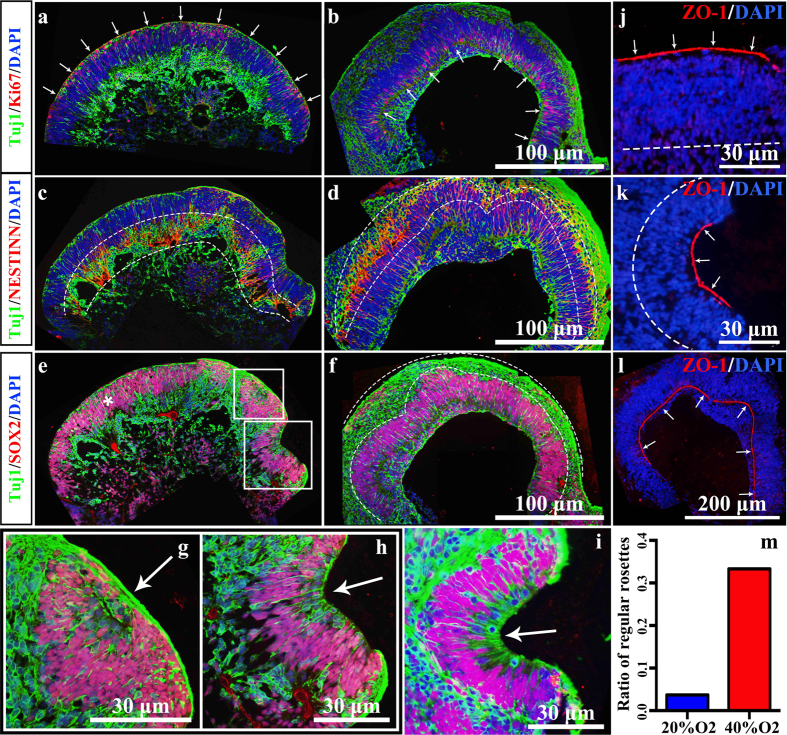
Cell proliferation within the NE requires oxygen support. (**a**, **c**, **e**) Tuj1 + Ki67, Tuj1 + NESTIN, Tuj1 + SOX2 double-stained NE in 62D 20% O_2_ group. (**b**, **d**, **f**) Tuj1 + Ki67, Tuj1 + NESTIN, Tuj1 + SOX2 double-stained in 50D 40% O_2_ group. White arrows in (**a**, **b**) point at the Ki67-positive proliferating cells in the NE of EB. The area between the two dotted lines in (**c**, **d**) represents the NESTIN-positive neural stem cells. The area between the two dotted lines in (**f**) represents the plate-ranged Tuj1-positive neurons. (**g**, **h**) Enlarged images of rosettes fusing at the edge of the EB in (**e**). (**i**) Enlarged Tuj1 (green) and SOX2 (red) double-stained images of typical rosettes fusing at the edge of EB in 50D 20% O_2_. White arrows in **g**, (**h**, **i**) point at the extroverting inner side of the rosettes. (**j**–**l**) ZO-1 staining of apical surface in NE. (**j**) An out-apical-side NE without forming rosettes. (**k**) NE forming the regular rosettes. (**l**) Developed rosette with an in-apical side. The dotted line in (**j**, **k**) represents the edge of the NE. White arrows in (**j, k**, **l**) point at the apical side. (**m**) Ratio of the developed in-apical regular rosettes in all 20% O_2_ groups and 40% O_2_ groups. NE: Neuroectodermal epithelium. EB: Embryonic body.

**Figure 7:**
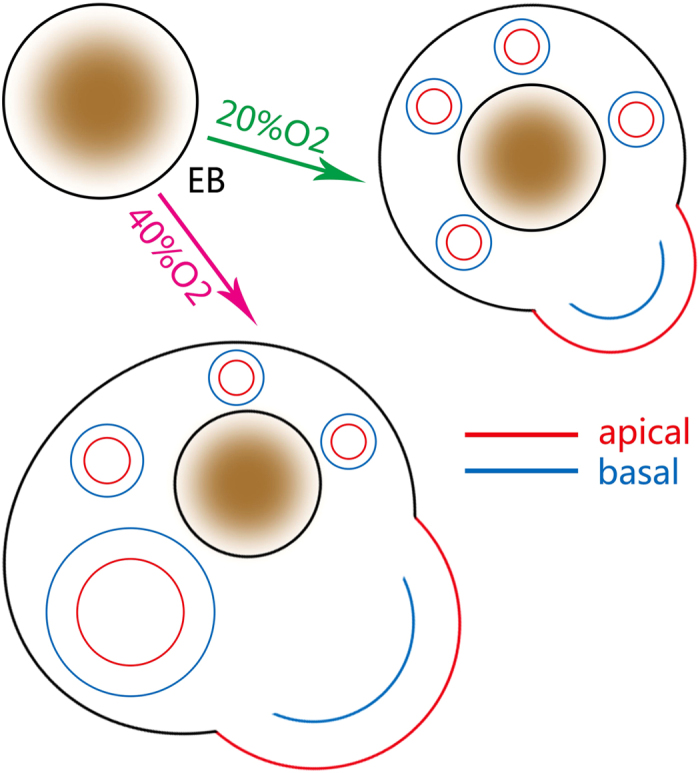
Summary of the effect on the development of neuroectodermal epithelium (NE) of intermittent high oxygen concentration. 1. Intermittent high oxygen concentration was able to generate a larger NE. 2. Under high oxygen concentration, neural rosettes could develop similar to *in vivo*, which was barely evident under normal oxygen concentrations. EB: embryonic body.

Under different oxygen concentrations, some differences in the formation of neural rosettes are observed. To test the different cellular distributions within NEs, we examined the mature neural marker Tuj1, the proliferation marker Ki67, the stem marker SOX2 and the neural stem cell (NSC) marker NESTIN (Fig. 6a–f). Simultaneously, because ZO-1 is expressed at the apical side of the NEs within neural rosettes^40^,^41^, we used ZO-1 staining to identify the apical membrane (Fig. 6j–l). Within the NEs, Ki67 positive cells were distributed at the apical side (outside) and the cell bodies of NESTIN positive cells were located mainly at the basal side (inside) (Fig. 6a, c). However, although these shaped continuous NEs could be seen in both the 20% O_2_ and 40% O_2_ groups, the NEs were relatively larger under 40% O_2_ (data not show). Once reformed from the NEs into the neural rosettes (Fig. 6g–i, k), these neural tube-like structures were not able to develop themselves under 20% O_2_ concentration and remained rosette-like (Fig. 6m). On the other hand, neural rosettes could grow under intermittent 40% O_2_ concentration and had identical *in vivo* apical-basal polarity with NEs (Fig. 6b, d, f, m). Thus, under intermittent high oxygen concentration, EBs could grow bigger and NEs could grow larger, compared to growth under normal oxygen concentration conditions. More important, formed neural rosettes could develop under intermittent high oxygen, which was barely evident under normal oxygen conditions (Fig. 7).

### Intermittent high oxygen concentration promoted the maturation and migration of retinal ganglion cells (RGCs) in NEs

**Figure 8:**
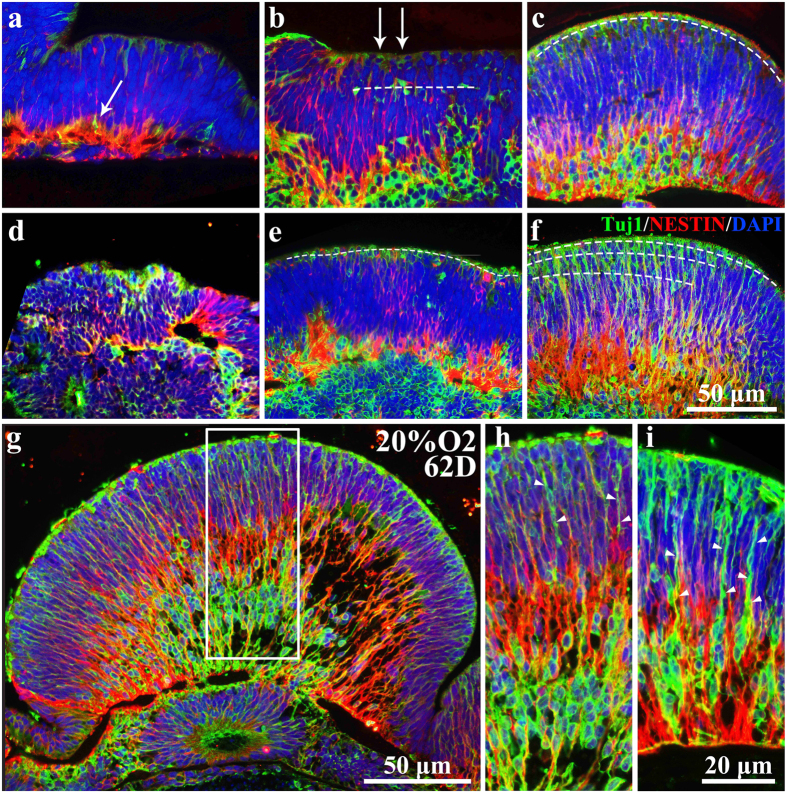
High oxygen concentration facilitated the proliferation, maturation and migration of RGCs in neuroectodermal epithelia of embryonic bodies. (**a**–**f**) Tuj1 and NESTIN double-stained NE in (**a**) 38D 20% O_2_; (**b**) 50D 20% O_2_; (**c**) 62D 20% O_2_; (**d**) 32D 40% O_2_; (**e**) 52D 20% O_2_; (**f**) 62D 40% O_2_. White arrows in (**a**,**b**) point at the Tuj1-positive mature neurons. The dotted lines in (**b**, **c**, **e**, **f**) delineate the distribution of Tuj1-positive mature neurons. (**g**) Whole Tuj1/NESTIN double-stained NE picture in 62D 20% O_2_ group. (**h**) Enlarged images in (**g**). (**i**) Regional image of Tuj1/NESTIN double-stained NE in 62D 40% O_2_ group. White arrows in (**h**, **i**) point at the process of mature neurons. NE: Neuroectodermal epithelium; RGCs: retinal ganglion cells.

Oxygen is a key component that controls the differentiated cellular fate of stem cells^23^. To determine the influence of oxygen concentration on RGCs, we used Tuj1 to mark these groups of cells^42^,^43^. RGCs induced *in vitro* were also generated at the ventricular margin (apical side), extended their basal processes and migrated to the basal side where the ganglion cell layer exited (Fig. 8a–i), as *in vivo*^42^,^43^. Simultaneously, Tuj1 positive RGCs located at the basal side also expressed the RGC marker Brn3 (Supplementary Fig. 3a–f). Using NESTIN to mark the NSCs, we found that the cell bodies of this group of cells were mainly located at the basal side of NEs with processes reaching the apical margin. Interestingly, Tuj1-positive RGCs elongated their processes and climbed towards the basal side with the processes of NESTIN-positive NSCs (Fig. 8g–i). This procedure was identical under both normal oxygen concentration and intermittent high oxygen concentration (Fig. 8a–f). However, the number of matured RGCs was significantly different between the two oxygen concentrations. On day 38, there were relatively few mature cells in the 20% O_2_ and 40% O_2_ groups. However, after intermittent high oxygen treatment, more mature neurons were evident (Fig. 8a, d). On day 50, fewer matured neurons were evident at the apical side in 20% O_2_ compared to 40% O_2_, where matured neurons ranged in line. On day 62, neurogenesis in the 20% O_2_ group was similar to the 40% O_2_ group on day 50 (Fig. 8c, e). However, neurogenesis in 40% O_2_ at this time point is much quicker, as the mature neurons arranged in multilayers within the NEs at the apical side (Fig. 8f). Meanwhile, the processes of Tuj1-positive neurons in 40% O_2_ on day 62 were longer and thicker than in 20% O_2_ at the same time point (Fig. 8g–i), illustrating that intermittent high oxygen treatment was able to promote the maturation of RGCs in NEs. Additionally, the migration of Tuj1-positive RGCs was improved under intermittent high oxygen concentration, as more mature RGCs could be seen in the middle of the NEs that were moving towards the basal side (Fig. 8c, f). In addition, NESTIN-positive NSCs implemented the scaffold function to let newly generated neurons move towards the basal side in all groups. Nevertheless, the relationship between RGCs and retinal stem cells was more compact in intermittent high oxygen conditions compared to normal oxygen conditions (Fig. 8c, f), which demonstrated that retinal stem cells were more functional and active under high oxygen environment.

## Discussion

It is a complex process to form an organ *in vitro* because different environmental conditions and local cell-cell interactions are involved^44^. For the enormous utility value of *in vitro*-derived organs, including regenerative medicine, tissue engineering and pharmacological testing, we must continue to generate tissues that mimic the *in vivo*-developed state. In this study, we confirmed the positive effect of intermittent high oxygen concentration on cell proliferation and differentiation within the hESCs-derived NR. Simultaneously, we found that adequate oxygen supply was essential for the normal neural development *in vitro* and the influence of the high oxygen on the development of RGCs was revealed. These findings suggested that intermittent high oxygen treatment, in addition to its convincing ability to promote cell proliferation and counteract apoptosis in ESCs^29^, might contribute to normal NR development *in vitro*.

In normal situations, aerobic metabolism is sustained by a sufficient oxygen supply^45^. We found that under high oxygen concentrations, the volume of EB increased significantly. The proliferation rate, as reflected by Ki67 staining, also demonstrated the productive effect of high oxygen concentrations. These consequences might result from the enhancement of aerobic metabolism under hyperoxic conditions. In this way, the increase in cell death under high oxygen concentration was in accordance with expectations. However, Because the retina is the highest oxygen consumption organ, it is critical to guarantee the oxygen level during retinal development^6^. Our results also showed that development modes marked by the specific retina-developmental marker PAX6^37^,^38^ were different under different oxygen concentrations. A high oxygen environment increased the distribution of PAX6 positive cells within the NR. It is possible that a high oxygen concentration promotes the differentiation of stem cells^25^. Or perhaps it is critical for the PAX6 gene to exert its function with the assistance of oxygen. As more PAX6 positive cells emerged within the NR, it was conceivable to say that adequate oxygen was essential for retinal development.

Neural development is accompanied by vascular development that transports essential oxygen and nutrients to the tissue. Because the neural tube is the origin of the nervous system, vascularization of the neural tube undoubtedly occupies an important position. This process is achieved by the perineural vascular plexus, which is located at both the inner and outer surface of the neural tube^46^. It is widely acknowledged that the *in vitro* inducement is the lack of cardiovascular system, which means that substance transportation proceeds via permeation. Because *in vitro* rosettes represent the early *in vivo* neural tube^17^,^18^,^19^, a guarantee of sufficient oxygen and nutrient supply is a crucial problem. In our study, we found that neural rosettes developed only under hyperoxic conditions. As the cell underwent mitosis at the inner surface, or apical side, of the neural tube, we contributed this to the adequate oxygen supply towards the inner surface from the outside liquid environment. Simultaneously, this result informed us that the oxygen concentration was not high enough for normal rosettes to grow via permeation under normoxic conditions. On the other hand, NR *in vitro* are avascularized. In addition, for retinal cell development, especially photoreceptors, a large number of mitochondria, responsible for high oxygen consumption, were found^47^. In this way, hyperoxic conditions may be essential for those *in vitro*-derived retinas that were not vascularized^29^.

The NR first forms a pseudo-stratified neuroepithelium that later differentiates into a layered structure with specific cellular types and positions. During the differentiating process, RGCs are the earliest neurons that undergo mitosis at the ventricular surface to reach their final destination^42^. In our study, we found that Tuj1-positive RGCs first emerged at the apical side of the NR and then migrated towards the basal side in all groups. Interestingly, these Tuj1-positive RGCs were migrating along the NESTIN-positive retinal stem cells located at the basal side. Under high oxygen concentrations, more Tuj1-positive RGCs were generated at the apical side and more migrating neurons were observed. One the one hand, these results suggested that *in vitro*-generated RGCs migrated along basal stem cells rather than traditional Müller cells. On the other hand, more importantly, as oxygen was supplied at the early stage of retinal development by the choroid plexus at the ventricular or apical surface *in vivo*^48^, both the generation and migration of RGCs seemed to be improved with a sufficient oxygen supply. Simultaneously, a longer process of Tuj1-positive RGCs and a tighter relationship of RGCs and NESTIN-positive retinal stem cells were evident under high oxygen conditions. Because hyperoxic conditions were of benefit for the differentiation of stem cells^24^,^25^,^26^,^27^, high oxygen concentrations could facilitate the growth, maturation and migration of RGCs.

## Materials and Methods

### hESC culture

The hESCs (H1 hESC line) were maintained using a feeder-free cultural protocol. In detail, hESCs were maintained in germfree 6-well plates (NEST, China) coated with 1% Matrigel (Corning, USA). The culture medium was changed daily with a mTeSR1 complete kit (STEMCELL Technologies, USA), a defined, feeder-free maintenance medium for hESCs. For passaging, hESCs were dissociated into single cells with TrypLE Express (Invitrogen, USA). After centrifugation (200 g for 3 min), hESCs were resuspended in mTeSR1 containing 10 μM Y-27632, which inhibits dissociation-induced apoptosis, and then planted in germfree 6-well plates at a density of 8000 cells per cm^2^.

### ESC identification

After stable passages, H1 cells were plated on glass slides and 6-well plates. To identify the embryonic characteristics of H1 cells, cellular immunofluorescence analysis as well as FACS were carried out as we previously described^49^,^50^. In brief, for immunostaining, cells were fixed with 4% paraformaldehyde (PFA) for 15 min at 4 °C followed by a 10-min rinse in 0.01 M phosphate buffer solution (PBS). After treatment with 0.3% Triton X-100 for 15 min and blocking with 3% bovine serum albumin (BSA) for 30 min at room temperature, the slides were incubated with the primary antibodies anti-SSEA4 (1:200; abcam, UK), anti-SOX2 (1:500; abcam), anti-NANOG (1:1000; abcam), or anti-OCT4 (1:500; abcam) in 1% BSA (overnight, 4 °C). The secondary antibodies cy3- or 488-conjugated (Jackson ImmunoResearch, West Grove, PA, USA) were then applied (1:200; 3 h). Before examination with a confocal laser-scanning microscope (Leica, Germany), sections were counterstained with 4′,6-diamidino-2-phenylindole (DAPI). For FACS analysis, H1 cells were digested by TrypLE Express (Invitrogen) for 3 min at 37 °C, followed by the addition of 3 ml 0.01 M PBS to stop digestion. After 300 g centrifugation for 3 min at 4 °C, H1 cells were resuspended in Stain Buffer (Biolegend, San Diego, CA). The specimens were then incubated with the fluorescence-conjugated antibodies anti-SSEA4 (BD Biosciences, AriaII, Franklin Lakes, NJ), anti-SOX2 (BD Biosciences), or anti-OCT4 (BD Biosciences) for 30 min on a shaker at 4 °C. After washing with wash buffer, H1 cells were then centrifuged at 200 g for 5 min at 4 °C. After removing the supernatant, 300 μl 0.01 M PBS was added to re-suspend the cells, which were then transferred to a flow cytometry tube for FACS analysis (BD Biosciences).

### Three dimensional culture to induce the optic cup formation

The 3-D induction was performed according to the previously described SFEBq culture^11^, with slight modifications. In detail, hESCs were dissociated into single cells in TrypLE Express (Invitrogen) containing 0.05 mg/ml DNase I (Roche, Switzerland) and 10 μM Y-27632 (Wako, Japan). After centrifugation and resuspension, 1 × 10^4^ single hESCs were reaggregated in a 96-well V-bottomed conical plate (PrimeSurface; Sumitomo Bakelite) with differentiation medium (G-MEM supplemented with 20% KSR, 0.1 mM nonessential amino acids, 1 mM pyruvate, 0.1 mM 2-mercaptoethanol, 100 U/mL penicillin, and 100 μg/mL streptomycin). Defining the day when the SFEBq culture was started as day 0, the wnt signal inhibitor IWR1e (Merck, USA) was added to the culture medium to a final 3 μM concentration from day 0 to day 12. Matrigel (final 1% v/v; growth-factor-reduced; BD Biosciences, USA) was added from day 2 to day 18. On day 6, half of the culture medium was replaced. On day 12, 96 aggregates were transferred to two identical dishes (10 cm in diameter) in the differentiation medium supplemented with 10% FBS (Gibco, USA). From day 15 to day 18, 3 μM CHIR99021 (R&D, USA) and 100 nM SAG (R&D) were added to the differentiation medium. After day 18, the whole aggregates were cultured in DMEM/F12-Glutamax medium (GIBCO) containing the N2 supplement (1×; Invitrogen), 10% FBS, 0.5 μM retinoic acid (Sigma-Aldrich), 0.25 μg/ml Fungizone (GIBCO), 100 U/ml penicillin, and 100 μg/ml streptomycin without Activin.

### Different oxygen supply

The cells were cultured in 5% CO_2_ incubators (Thermo, USA). To create a high oxygen environment, a sealed plastic box (248 mm × 180 mm × 150 mm) (LOCK&LOCK, Korea) with air scoops was put into one incubator. After day 18, two dishes were put in the other incubator and the boxes were labeled 20% O2 and 40% O2, respectively. In order to set up intermittent hyperoxic condition, mixed 40% oxygen air (40% O_2_ + 5% CO_2_ + 55% N2) was supplied to the box for 10 minutes each time, 3 times a day. These 3 intermittent high oxygen treatments were evenly distributed within 24 h and last daily from day 18 to day 62. To illustrate the development of NR more accurately, 3 time points were chosen: Day 38, 50, and 62.

### Measurement of the volume of embryonic bodies

Because hESCs aggregate to form embryonic bodies with optic vesicles and optic cups, it is not accurate to measure the volume of the embryonic bodies simply via their diameters. Considering the special shape of embryonic body, we divided it into two main parts: 1. an ellipsoid with a long axis (a) and two identical short axes (b) and 2. several (n: the number of protuberance) spheriform segments with a radius (rn) at the bottom and a height (hn) (Fig. 2g) . The volume of an EB would be expressed as follows:
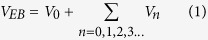
where V_0_ represents the volume of the ellipsoid and V_n_ represents the volume of the spheriform segments.

Using calculus, we calculated the volume of each embryonic body. Assume the equations of the ellipse and circle to be:
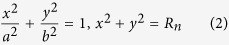
where “a” equals the length of long axis, “b” equals the length of short axes and “R_n_” equals the radius of sphere to which the spheriform segments belong. Then, we have:
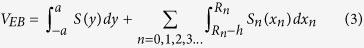

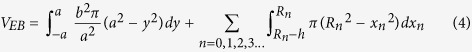

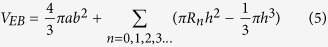


Because this is a triangle, we have:
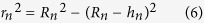


So the volume of the EB should be:
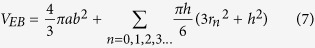


Considering the center of EBs to be an ellipsoid with two identical short axes, the volume of center could be calculated with equation (5). Since the density of dead cells within the center had been calculated, the number of total dead cells could be figured out.

### Fixation and section of EB

On days 38, 50 and 62, 3 embryonic bodies were randomly chosen from the 20% O_2_ group and the 40% O_2_ group. Three inductions were carried out, where 9 embryonic bodies (n = 9) from each group at each time point were chosen. To preserve a better tissue structure, paraffin imbedding was performed as previously described^51^. In, brief, after washing in 0.01 M PBS twice, EBs were put into 4% PFA in 0.01 M PBS for a 6-hour fixation. Fixed EBs were dehydrated with a graded series of ethanol and chloroform, and then embedded in paraffin wax with the NE paralleling to the section plane. Using a paraffin microtome, 5-μm thick paraffin sections were collected. After drying for 2 days at 37 °C, the sections were stored at room temperature.

### Immunofluorescence analysis

After being deparaffinized and rehydrated, the immunohistochemistry of sections was performed as we described previously^51^. Briefly, after treating with 0.3% Triton X-100 and 3% BSA and incubating at room temperature for 30 min, sections were incubated with the primary antibody anti-RAX (1:200; abcam, UK), anti-CHX10 (1:200; Santa Cruz; USA), anti-SOX2 (1:500; abcam), anti- β tubulin III (1:1000; Beyotime, Shanghai, China), anti-Ki67 (1:500; abcam) or anti-NESTIN (1:800; Thermo) in 1% BSA (overnight, 4 °C). The secondary antibodies cy3- or 488-conjugated (Jackson ImmunoResearch, West Grove, PA, USA) were then applied (1:200; 3 h). Before examination with a confocal laser scanning microscope (Leica, Germany), the sections were counterstained with DAPI (Sigma-Aldrich, St. Louis, MO, USA).

### Cell counts and Statistical analysis

#### Immunoreactive cells counting

In each group, at least three comparable EBs sections across the NE were stained with the neural progenitor marker PAX6 and proliferating cell marker Ki67. Cell number was determined using image J software (NIH, Bethesda, MD, USA). In detail, using a high-magnification microscope (400×), the images of the NEs were taken. Then, three 125 μm × 75 μm different rectangular areas with as large as possible NEs were chosen from each section and the number of PAX6 and Ki67 positive cells and the overall cells in the area were counted manually.

#### Regular Neural rosettes counting

We combined the 20% O2 group and 40% O2 together, ignoring the time points, into two groups: normoxia and hyperoxia. In each group, all immunofluorescence sections were selected. With DAPI staining of the nucleus, classical neural rosettes with the apical surface inside on each section were counted. Developed and undeveloped rosettes were recorded, where developed rosettes were defined according to the following aspects: 1. Rosettes showed a larger irregular or elliptical shape rather than the normal rounded shape; and 2. The diameter of the lumen formed by the apical surface was greater than 25 μm. With these two principal rules, the ratio of developed rosettes in the two groups was calculated.

#### Statistical analysis

Using the Statistical Product and Service Solutions software V17.0 (SPSS, Chicago, IL, USA), the cellular data were analyzed by one-way analyses of variance followed by Fisher’s protected least-significant difference post hoc tests. All data are expressed as the mean ± standard error. The significance level was set at 0.05.

## Additional Information

**How to cite this article**: Gao, L. *et al*. Intermittent high oxygen influences the formation of neural retinal tissue from human embryonic stem cells. *Sci. Rep.* **6**, 29944; doi: 10.1038/srep29944 (2016).

## Supplementary Material

Supplementary Information
