# Supplementary material for: Intermittent high oxygen influences the formation of neural retinal tissue from human embryonic stem cells
Source: Sci Rep. 2016 Jul 20;6:29944. doi: 10.1038/srep29944 (PMC4951725; doi:10.1038/srep29944)
Supplement: Supplementary Information [file srep29944-s1.pdf]

Supplementary Information

Intermittent high oxygen influences the formation of neural retinal tissue from human embryonic stem cells

Lixiong Gao[1, 2], Xi Chen[1, 2, 3, 4], Yuxiao Zeng[1, 2], Qiyou Li[1, 2], Ting Zou[1, 2], Siyu Chen[1, 2], Qian Wu[1, 2], Caiyun Fu[1, 2], Haiwei Xu[1, 2*], Zheng Qin Yin[1, 2*]

[1]Southwest Hospital/Southwest Eye Hospital, Third Military Medical University, Chongqing 400038, China

[2]Key Lab of Visual Damage and Regeneration & Restoration of Chongqing，Chongqing 400038, China

[3]School of Medicine, Nankai University, Tianjin 300071, China

[4]Department of Ophthalmology, Chinese People's Liberation Army General Hospital, Beijing 100853, China

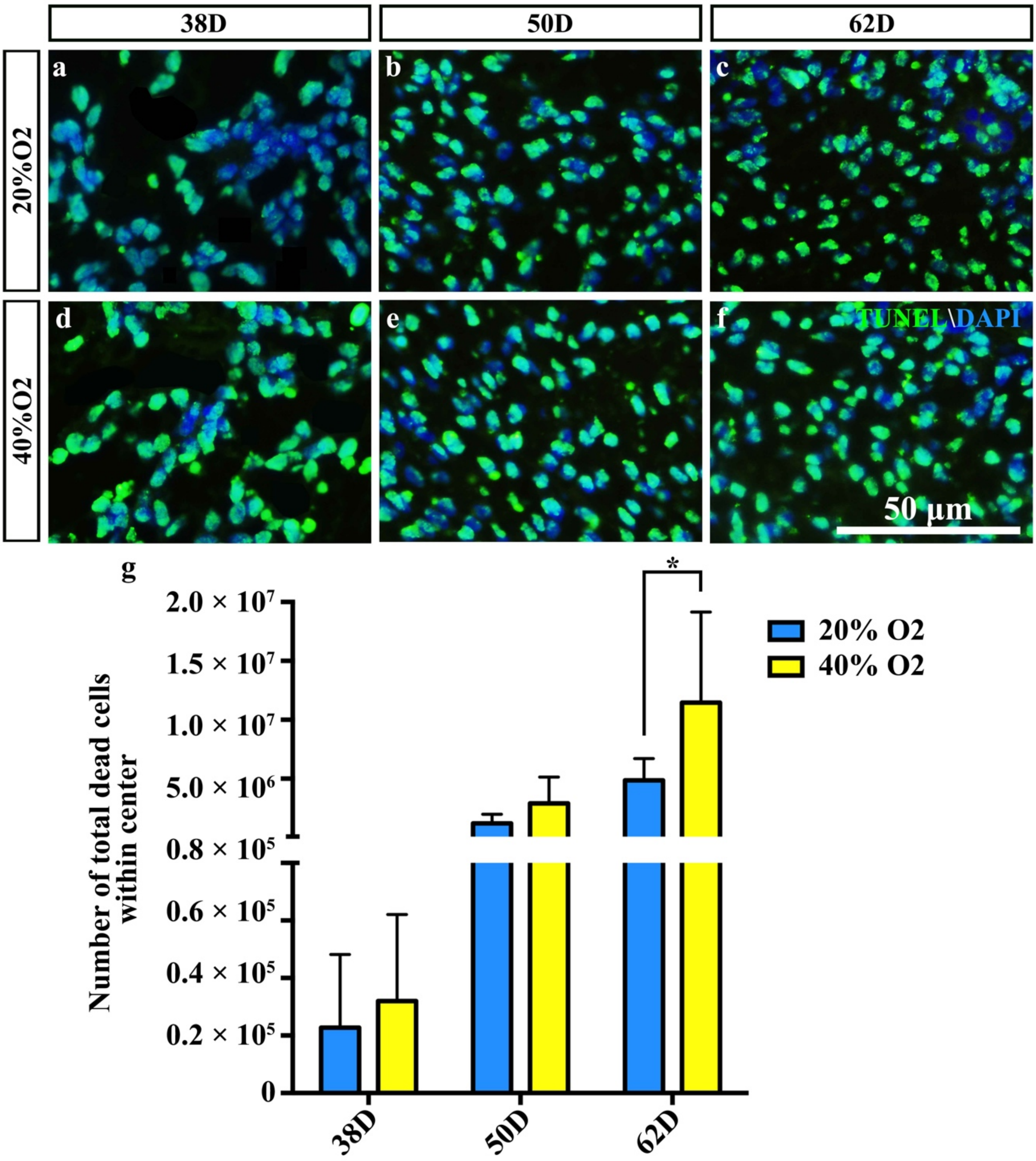

Supplementary figure 1 TUNEL staining showed central dead cells on long-term culture. **a**-**f**: TUNEL staining of central area in **a**: 38D 20% O2; **b**: 50D 20% O2; **c**: 62D 20% O2; **d**: 32D 40% O2; **e**: 52D 20% O2; **f**: 62D 40% O2. **g**: Number of total dead cells within the center.

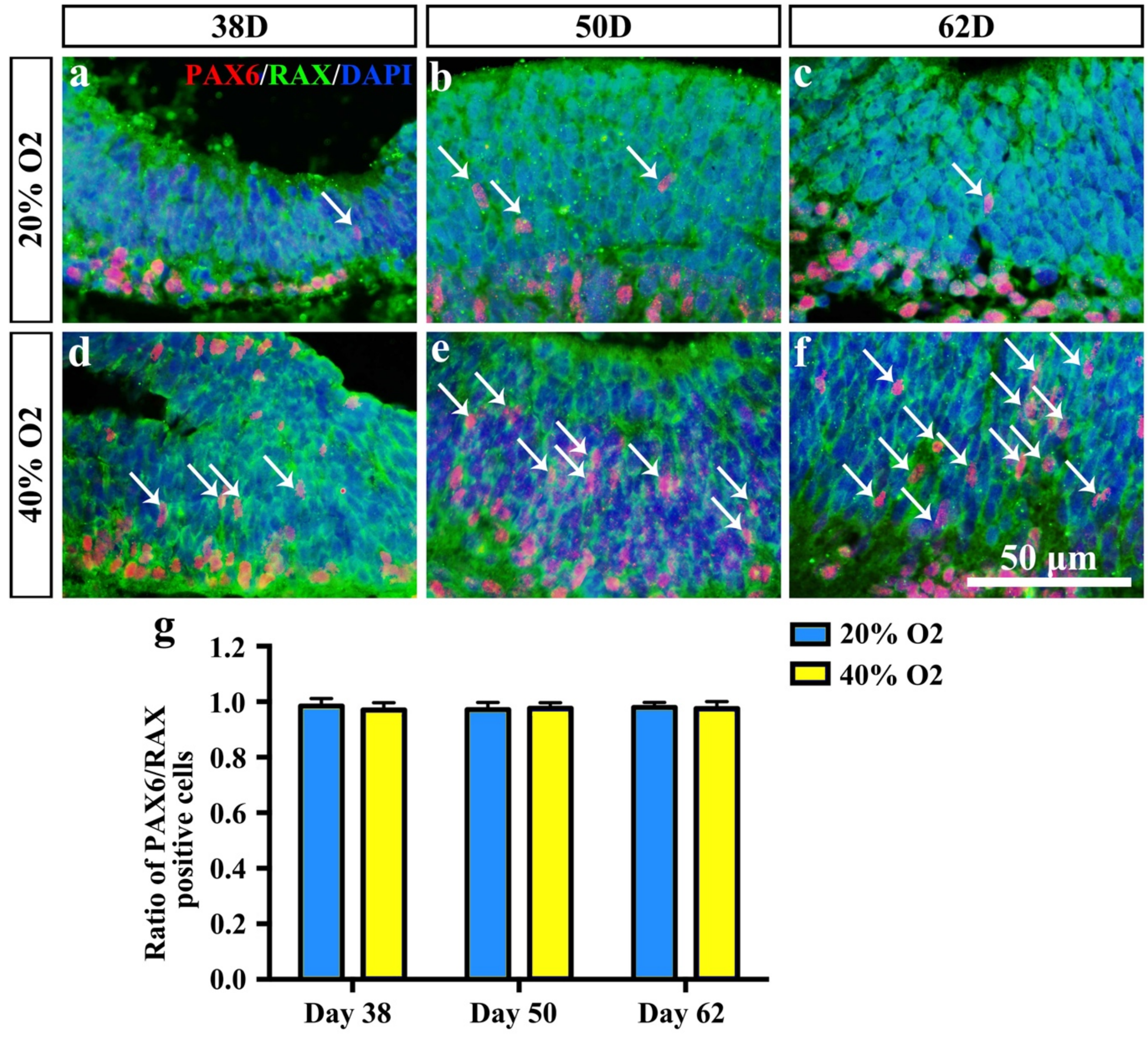

Supplementary figure 2 PAX6 positive cells expressed retinal cell marker RAX. **a-f**: PAX6 and RAX double-staining of NE in **a**: 38D 20% O2; **b**: 50D 20% O2; **c**: 62D 20% O2; **d**: 32D 40% O2; **e**: 52D 20% O2; **f**: 62D 40% O2. White arrows point at the PAX6-immunoreactive cells within the NE. **g**: Ratio of PAX6 and RAX-immunoreactive cells within the NE in 6 groups. Nearly all PAX6 positive cells expressed retinal cell marker RAX, which indicated that PAX6 positive neural progenitors are retinal progenitor cells. NE: Neuroectodermal epithelium.

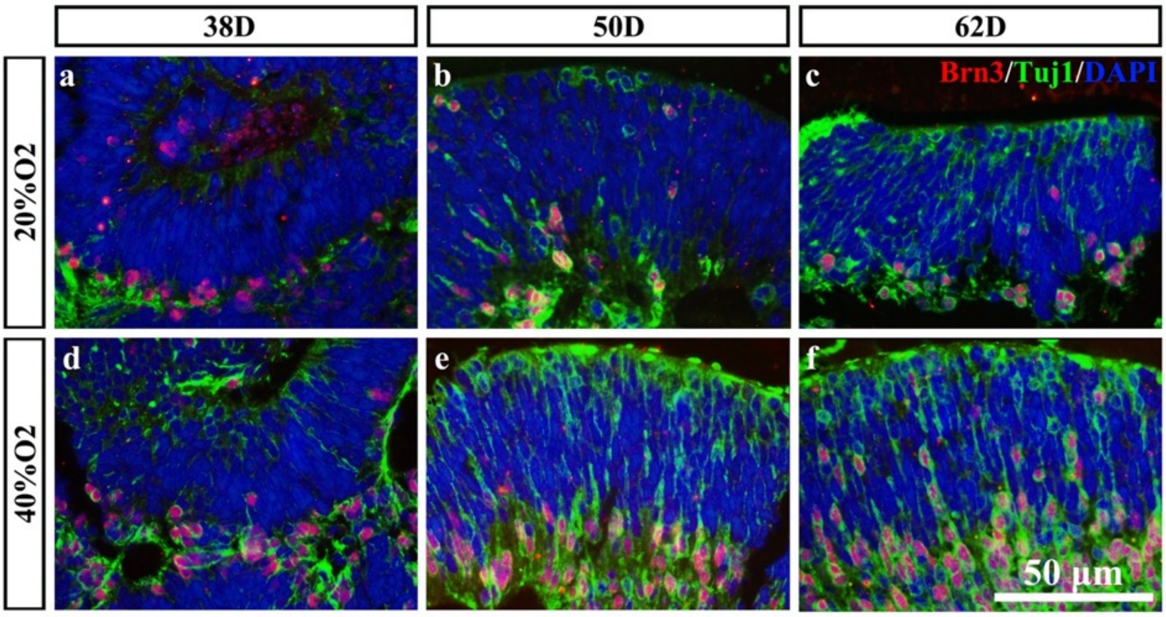

Supplementary figure 3 TUJ1 and BRN3 double staining showed Tuj1 positive cells are RGCs. **a-f**: TUJ1 and BRN3 double-staining of NE in **a**: 38D 20% O2; **b**: 50D 20% O2; **c**: 62D 20% O2; **d**: 32D 40% O2; **e**: 52D 20% O2; **f**: 62D 40% O2. NE: Neuroectodermal epithelium.

Supplementary method:

1 Terminal deoxynucleotidyl transferased UTP nick end labeling (TUNEL) staining

To test the apoptotic cells at the center of EBs, TUNEL assays were performed according to the instructions of the manufacturer with the In Situ Cell Death Detection Kit (Fluorescein or TMR, Roche Diagnostics, Germany). In brief, sections were incubated in a reaction mixture (Enzyme Solution 1 μL+Label Solution 9 μL) at 37°C for 1 h after the secondary antibody implementation. Following DAPI counterstaining and PBS washing, sections were viewed and photographed under a Zeiss (Oberkochen, Germany) Axiovert microscope equipped with a Zeiss Axio Cam digital color camera connected to the Zeiss Axio Vision 3.0 system. Dead cells were manually counted. Since the thickness of section is 5 μm, the average density of dead cells could be calculated.
